# Supplementary material for: Inactivation of Pol θ and C-NHEJ eliminates off-target integration of exogenous DNA
Source: Nat Commun. 2017 Jul 7;8:66. doi: 10.1038/s41467-017-00124-3 (PMC5501794; doi:10.1038/s41467-017-00124-3)
Supplement: Supplementary file 1 — Supplementary Information [file 41467_2017_124_MOESM1_ESM.pdf]

File name: Supplementary Information
Description: Supplementary figures.

File name: Supplementary Data 1
Description: Random integration junctions

File name: Supplementary Data 2
Description: Graphic representations of right-border junctions of random integration events

File name: Supplementary Data 3
Description: sgRNA and genotyping primer sequences for CRISPR/Cas9 knock-outs

File name: Supplementary Data 4
Description: Oligonucleotides used in the study

File name: Peer review file
Description:

a

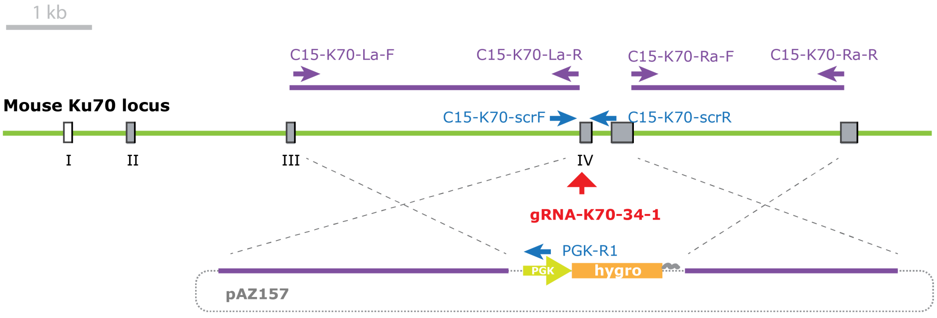

b

knockout cell-line	sequence	mutation	stop codon (amino acid)	truncated protein (% of WT protein)
Polq $^{-/-}$ #1	5' CGGCGGCGTTCATCGTCCGGCTCCGACACGTTCTCGGGAGATGGTGATAGCTTTGTTAGCCCTCAGCTCCG 3'	WT (exon 1, *Polq*)		
	CGGCGGCGTTCATCGTCCGGCTC-------------------------AGCTTTGTTAGCCCTCAGCTCCG	- 25bp	30	1.18%
	CGG---ATAGCTTTGTTAGCCCTCAGCTCCG	- 43bp	24	0.94%
Polq $^{-/-}$ #2	5' AATACCATAGTTTTGGTGTAAGAAAGATGTTCGAATGGCAGGCAGAATGCCTTTTGCTTGGTCACGTCTTGG 3'	WT (exon 2, *Polq*)		
	AATACCATAGTTTTGGTGTAA--------------------------TGCCTTTTGCTTGGTCACGTCTTGG	- 26bp	140	5.50%
	AATACCATAGTTTTGGTGTAAGAAAGATGTTC↓GAATGGCAGGCAGAATGCCTTTTGCTTGGTCACGTCTTGG<br>**C**	+ 1bp	150	5.90%
Ku80 $^{-/-}$ #1	5' GCTGTTGTGCTGTGTGTGGATGTGGGGGTTGCCATGGGTAACTCCTTTCCTGGTGAAGAATCTCCAATTGAA 3'	WT (exon 2, *Ku80*)		
	GCTGTTGTGCTGTGTGTGGATGTGGGGGT-------------------------GAAGAATCTCCAATTGAA	- 25 bp	29	3.96%
	GCTGTTGTGCTGTGTGTGGATGTGGGGGT-----↓----TAACTCCTTTCCTGGTGAAGAATCTCCAATTGAA<br>**AGCTGTTGTGCTGTGTGTGGATGTGTGGG**	- 9bp + 29bp	44	6.01%
Ku80 $^{-/-}$ #2	5' TGAAGTGTTCACTGACCTCAGCAGCCCGTTCAGCCAGGACCAACTGGACGTTATAATTTGTAACTTGAAGAA 3'	WT (exon 5, *Ku80*)		
	TGAAGTGTTCACTGACCTCAGCAGCCCGTT----CAGGACCAACTGGACGTTATAATTTGTAACTTGAAGAA	- 4bp	149	20.36%
	TGAAGTGTTCACTGACCTCAGCAGCCCGTTC-↓-CCAGGACCAACTGGACGTTATAATTTGTAACTTGAAGAA<br>**C**	- 2bp + 1bp	150	20.49%
LigIV $^{-/-}$ #1	5' TGATGCAGTTGAGTTGCACAACGTCACCACAGATCTGGAAAAGGTCTGCAGGCAGCTGCATGACCCCTCTGT 3'	WT (exon 1, *LigIV*)		
	TGATGCAGTTGAGTTGCACAACGTCACC----↓-----GAAAAGGTCTGCAGGCAGCTGCATGACCCCTCTGT<br>**TTTTCCA**	- 9bp + 7bp	235	25.80%
	TGATGCAGTTGAGTTGCACAACGTCACC-----------↓------------GCAGCTGCATGACCCCTCTGT<br>**TGCAGACCTTTTCCAG**	- 23bp + 16bp	230	25.25%
LigIV $^{-/-}$ #2	5' TTCTCCAGAAACGGTTACAACTATACCGACCAGTTTGGTGAATCTCCACAGGAAGGCTCTCTCACCCCATTT 3'	WT (exon 1, *LigIV*)		
	TTCTCCAGAAACGGTTACAACTATACCGACCA-↓-TTGGTGAATCTCCACAGGAAGGCTCTCTCACCCCATTT<br>**TGGTGA**	- 2bp + 6bp	305	33.48%
	TTCTCCAGAAACGGTTACAACTAT------↓-------GTGAATCTCCACAGGAAGGCTCTCTCACCCCATTT<br>**TACAACTGAGTATG**	- 13bp + 14bp	301	33.04%
Polq $^{-/-}$ *Ku80* $^{-/-}$ #1	5' AATACCATAGTTTTGGTGTAAGAAAGATGTTCGAATGGCAGGCAGAATGCCTTTTGCTTGGTCACGTCTTGG 3'	WT (exon 2, *Polq*)		
	AATACCATAGTTTTGGTGTAAGAAAGAT----------CAGGCAGAATGCCTTTTGCTTGGTCACGTCTTGG	- 10bp	107 (*Polq*)	4.21%
	AATACCATAGTTTTGGTGTAAGAAAGATGTTC↓GAATGGCAGGCAGAATGCCTTTTGCTTGGTCACGTCTTGG<br>**C**	+ 1bp	150 (*Polq*)	5.90%
Polq $^{-/-}$ *Ku80* $^{-/-}$ #2	5' AATACCATAGTTTTGGTGTAAGAAAGATGTTCGAATGGCAGGCAGAATGCCTTTTGCTTGGTCACGTCTTGG 3'	WT (exon 2, *Polq*)		
	AATACCATAG---ACGTCTTGG	- 53bp	132 (*Polq*)	5.19%
	AATACCATAGTTTTG---CTTGGTCACGTCTTGG	- 41bp	136 (*Polq*)	5.35%
Polq $^{-/-}$ *LigIV* $^{-/-}$ #1	5' AATACCATAGTTTTGGTGTAAGAAAGATGTTCGAATGGCAGGCAGAATGCCTTTTGCTTGGTCACGTCTTGG 3'	WT (exon 2, Polθ)		
	AATACCATAGTTTTGGTGTA-----------------------AGAATGCCTTTTGCTTGGTCACGTCTTGG	- 23bp	142 (*Polq*)	5.58%
	AATACCATAGTTTTGGTGTA-----------------------AGAATGCCTTTTGCTTGGTCACGTCTTGG	- 23bp	142 (*Polq*)	5.58%
Polq $^{-/-}$ *LigIV* $^{-/-}$ #2	5' AATACCATAGTTTTGGTGTAAGAAAGATGTTCGAATGGCAGGCAGAATGCCTTTTGCTTGGTCACGTCTTGG 3'	WT (exon 2, Polθ)		
	AATACCATAGTTTTGGTGTAAGAAAGA-------ATGGCAGGCAGAATGCCTTTTGCTTGGTCACGTCTTGG	- 7bp	108 (*Polq*)	4.25%
	AATACCATAGTTTTGGTGTAAGAAAGA-------------------ATGCCTTTTGCTTGGTCACGTCTTGG	- 19bp	104 (*Polq*)	4.09%

Supplementary Figure 1. Generation and characterization of the knock-out ES cells used in this study. a, Strategy for CRISPR-Cas9-stimulated gene targeting of the *Ku70* (Xrcc6) gene in mouse ES cells. **b,** Sequences of the CRISPR-Cas9-induced mutations in *Ku80* (Xrcc5), *LigIV* and *Polq* genes, and the predicted changes to the protein resulting from them.

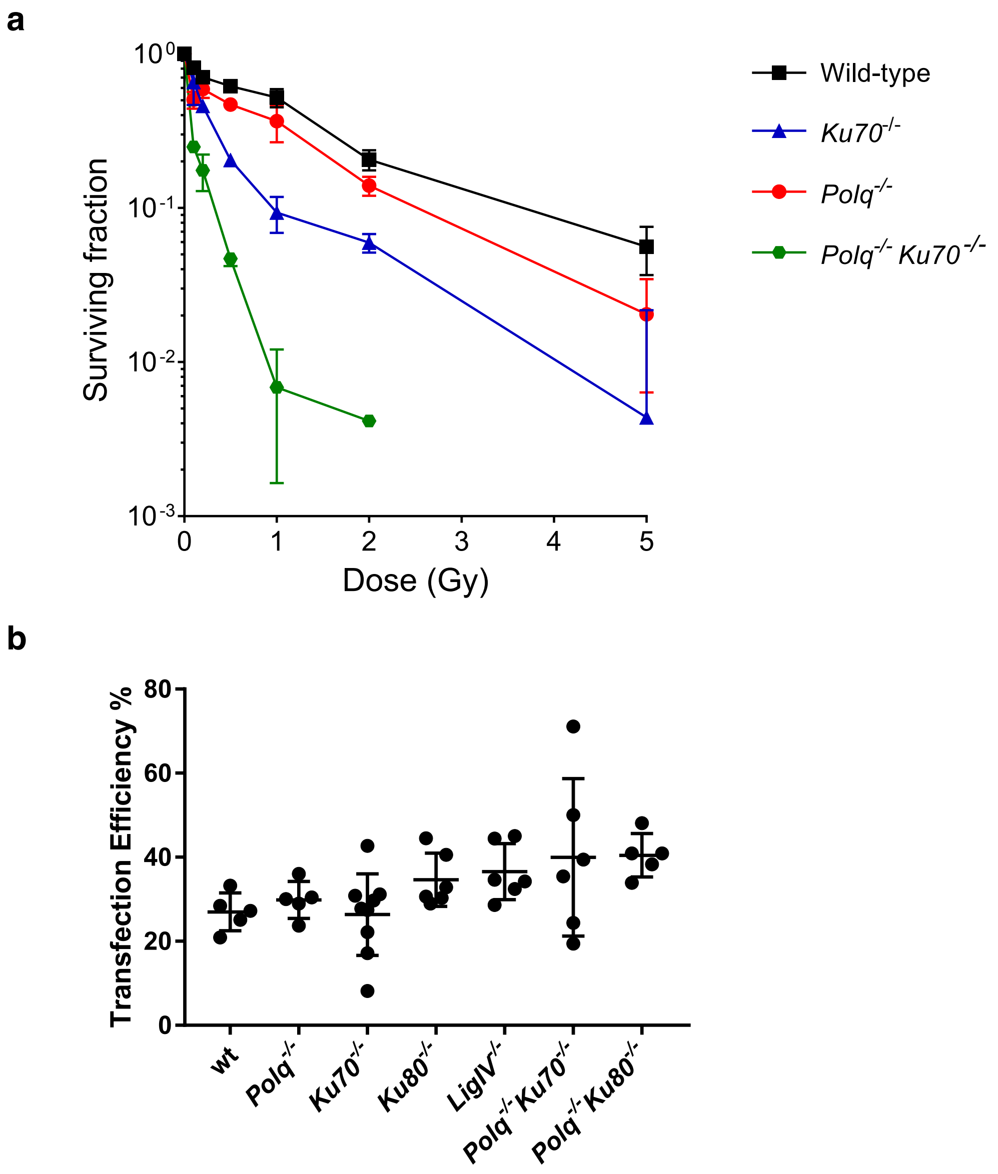

Supplementary Figure 2. a, Ionising radiation sensitivity of the knockout ES cell lines determined by clonogenic survival. Cells were irradiated with different doses immediately after plating. Data shown are the mean ± SEM (n = 3) **b,** Efficiency of transfection by electroporation of the knockout ES lines was determined by measuring the expression of GFP from circular reporter plasmid co-transfected with the RI plasmid DNA.

a

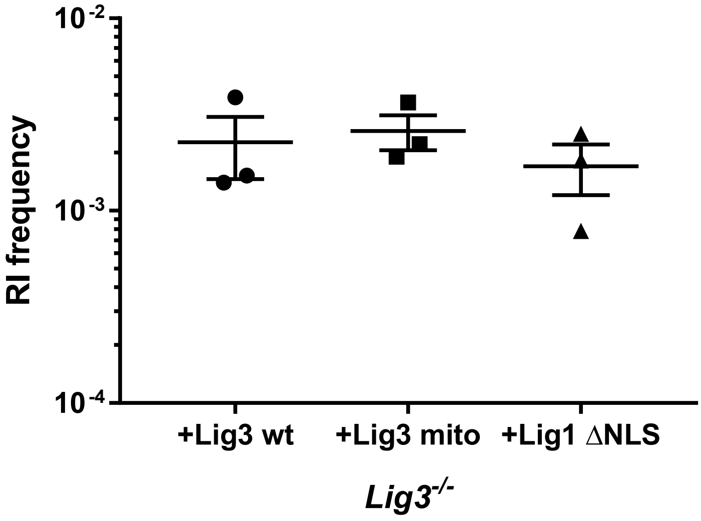

b

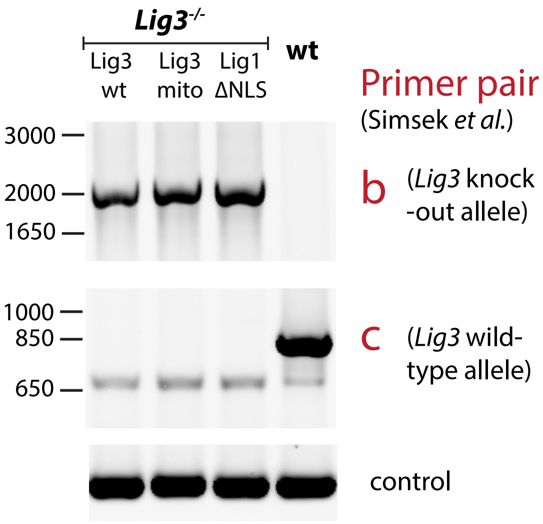

Supplementary Figure 3. RI frequency is not affected by nuclear Lig3 deficiency. a, *Lig3* knock-out cells preemptively complemented with wild-type Lig3, mitochondrial-exclusive form of Lig3, or Lig1-GFP lacking nuclear localization signal, to overcome the lethality of mitochondrial Lig3 deficiency[24], were transfected with linearized puromycin resistance construct (pLPL) and RFP-expressing construct for transfection efficiency estimation; RI frequency (number of puromycin-resistant colonies formed after 8 days of selection adjusted for plating and transfection efficiencies) in three independent experiments is plotted. **b.** Absence of wild-type *Lig3* allele was verified by PCR genotyping.

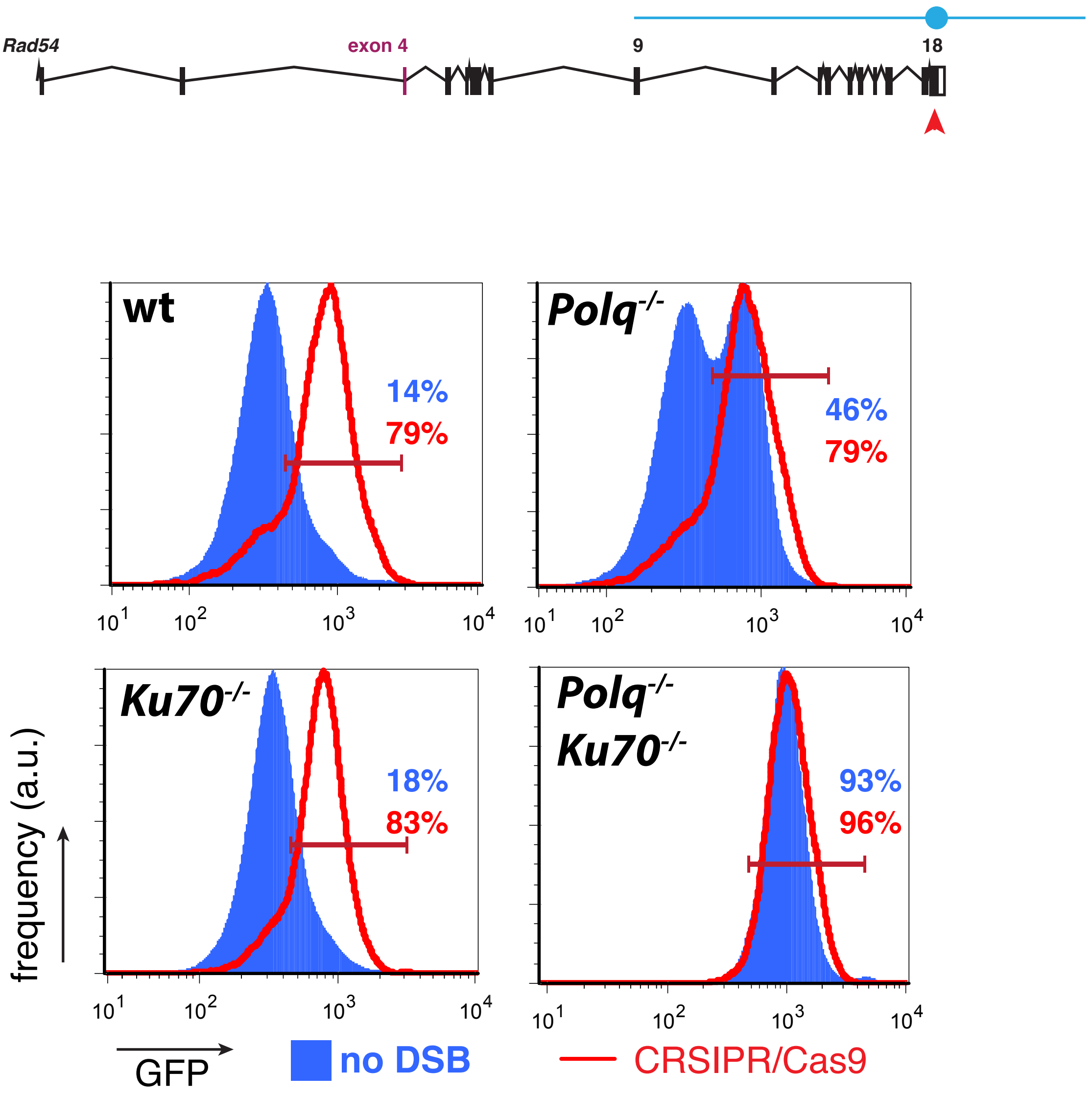

Supplementary Figure 4. CRISPR-Cas9-stimulated gene targeting in C-NHEJ and TMEJ-deficient ES cells. Construct targeting *Rad54* exon 18, which in wild-type cells recombines at low frequency, was used to measure the effect of *Polq* and *Ku70* deficiency, and CRISPR-Cas9 DSB induction on gene targeting. DSB was induced in the *Rad54* region replaced by GFP-puro cassette (red arrow on the locus scheme) by co-transfection of the gRNA-Cas9 expression construct with the circular gene targeting construct. Representative FACS frequency plots from three independent experiments are shown.

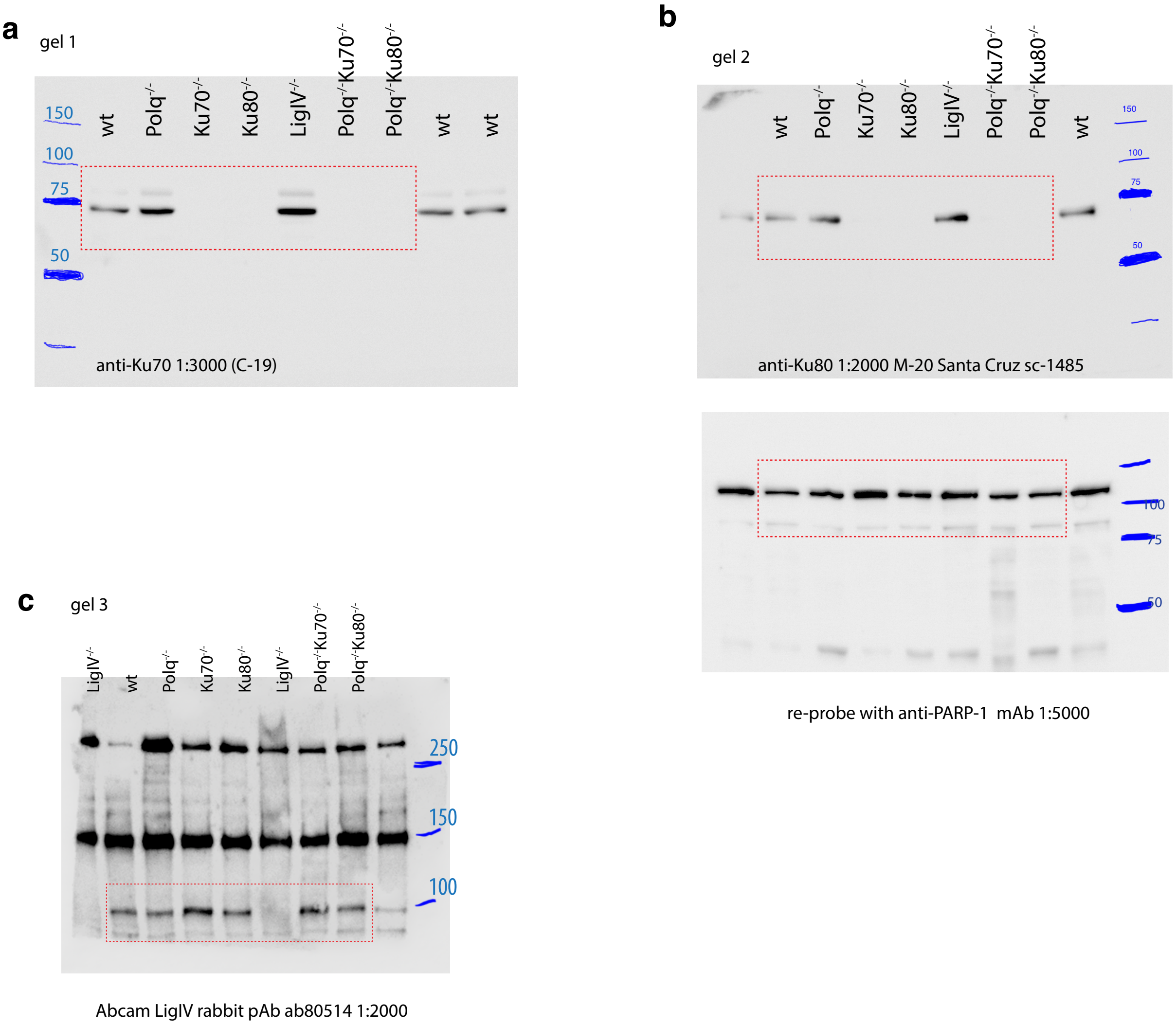

Supplementary Figure 5. Uncropped scans of the immunoblots shown in Fig 1a. Regions shown in Fig. 1a are indicated with dotted red box.
