# Supplementary material for: Inactivation of Pol θ and C-NHEJ eliminates off-target integration of exogenous DNA
Source: Nat Commun. 2017 Jul 7;8:66. doi: 10.1038/s41467-017-00124-3 (PMC5501794; doi:10.1038/s41467-017-00124-3)
Supplement: Supplementary file 3 — Supplementary Data 2 [file 41467_2017_124_MOESM3_ESM.docx]

**Supplementary Data 2. Right-border junctions of random integration events**

**Without insertions** Plasmid-Mouse genome

Wild type ATAAAGTGTAAAGCCTGGGG**TGC**TGTCTTTCTTCCACTGGAT Junction

ATAAAGTGTAAAGCCTGGGG**TGC**CTAATGAGTGAGCTAACTC Plasmid

CCAGCACCAATTGTTGAAAA**TGC**TGTCTTTCTTCCACTGGAT Chr 1

Wild type ACAACATACGAGCCGGAAGCA**TA**TCCCTTGGGAATTCTGAAT Junction

ACAACATACGAGCCGGAAGCA**TA**AAGTGTAAAGCCTGGGGTG Plasmid

TGGCGTTATCTGATCATTTTC**TA**TCCCTTGGGAATTCTGAAT Chr 2

Wild type GCCTAATGAGTGAGCTAACTC**A**GGTTTCACCATATTATATTC Junction

GCCTAATGAGTGAGCTAACTC**A**CATTAATTGCGTTGCGCTCA Plasmid

TGAAATTTGGGAAATCTGAAA**A**GGTTTCACCATATTATATTC Chr 2

Wild type GCCCAGACCCTTGCCCTGGT**GGT**CACACCTCCAAATGGTGCT Junction

GCCCAGACCCTTGCCCTGGT**GGT**CGGGCGAGACGCCGACGGT Plasmid

GGTCACACCTATTCTAACAA**GGT**CACACCTCCAAATGGTGCT Chr 3

Wild type TAATTGCGTTGCGCTCACTG**CCC**CATACCCAAGTACTTAACC Junction

TAATTGCGTTGCGCTCACTG**CCC**GCTTTCCAGTCGGCTTAAT Plasmid

CCAATGCTATACCAAAAGTC**CCC**CATACCCAAGTACTTAACC Chr 4

Wild type CATACGAGCCGGAAGCATAAA**G**GTAATTACTTTGGGGACACA Junction

CATACGAGCCGGAAGCATAAA**G**TGTAAAGCCTGGGGTGCCTA Plasmid

CCAGCCACCTCTTCAATCGGC**G**GTAATTACTTTGGGGACACA Chr 5

Wild type AGCTGTTTCCTGTGTGAAATT**G**GTCAGTTGTTTTGTCTTGTT Junction

AGCTGTTTCCTGTGTGAAATT**G**TTATCCGCTCACAATTCCAC Plasmid

AGATTACAGGCAAGCCTCAAC**G**GTCAGTTGTTTTGTCTTGTT Chr 7

Wild type AAGGCCCGGAGATGAGGAAGA**G**GCCAGCGGCGAGCAGTTTCT Junction

AAGGCCCGGAGATGAGGAAGA**G**GAGAACAGCGCGGCAGACGT Plasmid

CGGAGGCCCCAGACCAGTGGC**G**GCCAGCGGCGAGCAGTTTCT Chr 11

Wild type ACGAGCCGGAAGCATAAAGT**GTA**CGCTCTGAAGTCCCAGGTG Junction

ACGAGCCGGAAGCATAAAGT**GTA**AAGCCTGGGGTGCCTAATG Plasmid

ACCTTCACCCCCGTGATGCC**GTA**CGCTCTGAAGTCCCAGGTG Chr 12

Wild type ACAACATACGAGCCGGAAGCA**T**TATTCTTCACTATGGGACAG Junction

ACAACATACGAGCCGGAAGCA**T**AAAGTGTAAAGCCTGGGGTG Plasmid

GTCATTGTGTTGTGTTTACTC**T**TATTCTTCACTATGGGACAG Chr 15

Wild type GAGCTAACTCACATTAATTGC**G**GCCGACGCCGCCATGAGCGC Junction

GAGCTAACTCACATTAATTGC**G**TTGCGCTCACTGCCCGCTTT Plasmid

CCCAACTTGGACGCCAGGTCC**G**GCCGACGCCGCCATGAGCGC Chr 16

Wild type TAACTCACATTAATTGCGTTGCTCTCCTGCTAGGTACAATAT Junction

TAACTCACATTAATTGCGTTGCGCTCACTGCCCGCTTTCCAG Plasmid

TACATTCATTCCCGACATCGTATCTCCTGCTAGGTACAATAT Chr 17

Wild type AATGAGTGAGCTAACTCACATTCTGTAGCTGTCTTCAGACAC Junction

AATGAGTGAGCTAACTCACATTAATTGCGTTGCGCTCACTGC Plasmid

ATATATATATTATATGAGTACACTGTAGCTGTCTTCAGACAC Chr X

Polq-/- AGCTGTTTCCTGTGTGAAAT**TGT**CTTTTTCCTGAAGACCTAC Junction

AGCTGTTTCCTGTGTGAAAT**TGT**TATCCGCTCACAATTCCAC Plasmid

AATCCTGTTTTGTACGGCAG**TGT**CTTTTTCCTGAAGACCTAC Chr 1

Polq-/- TCCGCTCACAATTCCACACA**ACA**CCGGTCTGAACTTCCATTC Junction

TCCGCTCACAATTCCACACA**ACA**TACGAGCCGGAAGCATAAA Plasmid

GCCCTCTGCCAAGCCTGGAG**ACA**CCGGTCTGAACTTCCATTC Chr 1

Polq-/- ATTGCGTTGCGCTCACTGCCC**GC**CCGGAGGTTCCCAAGGCGG Junction

ATTGCGTTGCGCTCACTGCCC**GC**TTTCCAGTCGGCTTAATGC Plasmid

TATTTATAATGGGGGTGAGTG**GC**CCGGAGGTTCCCAAGGCGG Chr 2

Polq-/- GACGCGCGTGAGGAAGAGTT**CTT**GAGGACCCAGGTTTGAGTT Junction

GACGCGCGTGAGGAAGAGTT**CTT**GCAGCTCGGTGACCCGCTC Plasmid

GTTAAGAGCACAGGCTGCTC**CTT**GAGGACCCAGGTTTGAGTT Chr 3

Polq-/- AGTGAGCTAACTCACATTAAT**T**ACAGGCCACCTGGATAGAAG Junction

AGTGAGCTAACTCACATTAAT**T**GCGTTGCGCTCACTGCCCGC Plasmid

GGACAGGTACCTCTGTACATG**T**ACAGGCCACCTGGATAGAAG Chr 3

Polq-/- GCCATGGCGGCCGGGAGCATG**C**GGCCGCTTAGTTCTACGGGT Junction

GCCATGGCGGCCGGGAGCATG**C**GACGTCGGGCCCAATTCGCC Plasmid

AGGGGTCCTCTTCCACCATTA**C**GGCCGCTTAGTTCTACGGGT Chr 4

Polq-/- ACAACATACGAGCCGGAAGCA**T**GCTGGGTAACATCAGGGCTA Junction

ACAACATACGAGCCGGAAGCA**T**AAAGTGTAAAGCCTGGGGTG Plasmid

TCCATAGTCTGCACCTTTATT**T**GCTGGGTAACATCAGGGCTA Chr 4

Polq-/- CTCACATTAATTGCGTTGCGCTGAGTTCCAGGCAGTTGTGAA Junction

CTCACATTAATTGCGTTGCGCTCACTGCCCGCTTTCCAGTCG Plasmid

GCATCGGAGCCTTCTGAAGCTAGAGTTCCAGGCAGTTGTGAA Chr 4

Polq-/- CCGCTCACAATTCCACACAAC**A**GGTTTTAAAACAAGAATAAG Junction

CCGCTCACAATTCCACACAAC**A**TACGAGCCGGAAGCATAAAG Plasmid

TATGCATAAAATAATTAATAA**A**GGTTTTAAAACAAGAATAAG Chr 4

Polq-/- GGTGCCTAATGAGTGAGCTAA**CT**GTAGTCCACACTCTCACCT Junction

GGTGCCTAATGAGTGAGCTAA**CT**CACATTAATTGCGTTGCGC Plasmid

GTCACAGAAGCTGTGTAGCTT**CT**GTAGTCCACACTCTCACCT Chr 5

Polq-/- ACATTAATTGCGTTGCGCTCA**CT**ACCATCTCCATGTATAAAT Junction

ACATTAATTGCGTTGCGCTCA**CT**GCCCGCTTTCCAGTCGGCT Plasmid

GCTCCCGGCTCACCTTCTTTC**CT**ACCATCTCCATGTATAAAT Chr 6

Polq-/- CATTAATTGCGTTGCGCTCA**CTG**GGACCCAAATAAGACTTTT Junction

CATTAATTGCGTTGCGCTCA**CTG**CCCGCTTTCCAGTCGGCTT Plasmid

CCCCTTATTTGAGCTACATC**CTG**GGACCCAAATAAGACTTTT Chr 7

Polq-/- GCTCACTGCCCGCTTTCCAGT**C**CCTCCCTCCCCTCCATGCAT Junction

GCTCACTGCCCGCTTTCCAGT**C**GGCTTAATGCGCCGCTACAG Plasmid

CCCACCCTCCTCTCCGTCCTT**C**CCTCCCTCCCCTCCATGCAT Chr 10

Polq-/- GCGCTCACTGCCCGCTTTCCAGCATGGATACAGACCCACTGC Junction

GCGCTCACTGCCCGCTTTCCAGTCGGCTTAATGCGCCGCTAC Plasmid

CAGGCTGCTCACAGCCACAGTCCATGGATACAGACCCACTGC Chr 11

Polq-/- AAGCCTGGGGTGCCTAATGA**GTG**GGATTGAGAAGAAAAACAC Junction

AAGCCTGGGGTGCCTAATGA**GTG**AGCTAACTCACATTAATTG Plasmid

ACAAGTTCTGAGTTTCTGTG**GTG**GGATTGAGAAGAAAAACAC Chr 11

Polq-/- AAGCATAAAGTGTAAAGCCTGGCCAACACCCATGAGGCAGCT Junction

AAGCATAAAGTGTAAAGCCTGGGGTGCCTAATGAGTGAGCTA Plasmid

CCAGAGGACTCTGGTTGGATTTCCAACACCCATGAGGCAGCT Chr 13

Polq-/- CCTGGGGTGCCTAATGAGTGAGAAGTTTCAATTCTACCACAG Junction

CCTGGGGTGCCTAATGAGTGAGCTAACTCACATTAATTGCGT Plasmid

ACCACCGTGCCTGGCTTGGCTAAAGTTTCAATTCTACCACAG Chr 17

Polq-/- TTAATTGCGTTGCGCTCACTG**C**TTTGACACATCAAGGACGTC Junction

TTAATTGCGTTGCGCTCACTG**C**CCGCTTTCCAGTCGGCTTAA Plasmid

CTCTGCAGCCGGTGGGAGCAT**C**TTTGACACATCAAGGACGTC Chr 18

Polq-/- ACTGTTGGGAAGGGCGATCGG**T**CAGCAAGTACTTGTTGACAT Junction

ACTGTTGGGAAGGGCGATCGG**T**GCGGGCCTCTTCGCTATTAC Plasmid

TGATGTTAAAAGTAGTTCCTT**T**CAGCAAGTACTTGTTGACAT Chr X

**With insertions** Plasmid-**insert-**Mouse genome

Wildtype Chr 1 Primed RC

ATAGCTGTTTCCTGTGTGAAATTGT**GAGCGGATAACGAGATATCTGACCT**TGACCTTGACCTGGGACTGGGGGAG

ATAGCTGTTTCCTGTGTGAAATTGTTATCCGCTCACAATTCCACACAACATACGAGCCGGAAGCATAAAGTGTAA

CTCAAACTGAGGAAGGCTCCGTCTTCCACGGCAGTGCCTCACGAGATATCTGACCTTGACCTGGGACTGGGGGAG

Wildtype Chr 1 Unknown

CGGAAGCATAAAGTGTAAAGCCTGGGGTGCCTAATGAG**CG**AGCCTGAGACAGGAGCACGGGGTTAAGGCTAAGTCC

CGGAAGCATAAAGTGTAAAGCCTGGGGTGCCTAATGAGTGAGCTAACTCACATTAATTGCGTTGCGCTCACTGCCC

AGTCACACTGTTGCTGGGCTGGATGAGCCAGCAGTGAGCAAGCCTGAGACAGGAGCACGGGGTTAAGGCTAAGTCC

Wildtype Chr 2 Unknown

GTCGGGCTCGACATCGGCAAGGTGTGGGTCGCGGACGA**GTGT**GTGTGTGTGTGTGTACATATATATATGTGTGTAT

GTCGGGCTCGACATCGGCAAGGTGTGGGTCGCGGACGACGGCGCCGCGGTGGCGGTCTGGACCACGCCGGAGAGCG

ACTATATATATATATATATATATATATATATATATATATATAGTGTGTGTGTGTGTACATATATATATGTGTGTAT

Wildtype Chr 2 Unknown

ACATTAATTGCGTTGCGCTCACTGCCCGCTTTCCAGTCG**G**TCTAGAGATGGGAGCAAAGTGTTCCTCATCCTAGGC

ACATTAATTGCGTTGCGCTCACTGCCCGCTTTCCAGTCGGCTTAATGCGCCGCTACAGGGCGCGTCCATTCGCCAT

CCTCCCCCGGCATTCCTGGAAATGAAGGGTGGGAGTCTCTTCTAGAGATGGGAGCAAAGTGTTCCTCATCCTAGGC

Wildtype Chr 3 Primed RC

CTAACTCACATTAATTGCGTTGCG**GCTTAATGGGCAA-/56/-CCCTTGCCCTGGT**GGTCACACCTCCAAATGGT

CTAACTCACATTAATTGCGTTGCGCTCACTGCCCGCTTTCCAGTCGGCTTAATG

ACTCCAACCAGGTCACACCTATTCTAACAAGGTCACACCTCCAAATGGT

Wildtype Chr 4 Primed RC

CGGAAGCATAAAGTGTAAAGCCTGGGGTGCCTAATG**TGAGTT**AGCTTTCCCTTTCTTACTACCCCAGCTGACTGAG

CGGAAGCATAAAGTGTAAAGCCTGGGGTGCCTAATGAGTGAGCTAACTCACATTAATTGCGTTGCGCTCACTGCCC

CACAGTTGGAGCCTTATTTAGCTTCTTTCCGGGTCGTCCGGAAGCTTTCCCTTTCTTACTACCCCAGCTGACTGAG

Wildtype Chr 4 Primed RC

CCTAATGAGTGAGCTAACTCACATTAATTGCGTTGCG**G**GCAATTTAACAGAAAGAGACAGCCTGTGTGCTGGGAG

CCTAATGAGTGAGCTAACTCACATTAATTGCGTTGCGCTCACTGCCCGCTTTCCAGTCGGCTTAATGCGCCGCTA

TATATGACCCTGAAAGCACCGCCTGCCAAGCAAAGGAAGCAATTTAACAGAAAGAGACAGCCTGTGTGCTGGGAG

Wildtype Chr 4 Unknown

TGTAAAGCCTGGGGTGCCTAAT**AAACACATGAAAC-/186/-TAGCAAGATTTTA**TCAACCACATTTTTTTAATCC

TGTAAAGCCTGGGGTGCCTAATGAGTGAGCTAACTCACATTAATTGCGTTGCGCTCACTGCCCGCTTTCCAG

GGCTGGTTTTGATGCTCTTCCTGGTATTAAATGAAATGTTTTCTTTTCATTCAACCACATTTTTTTAATCC

Wildtype Chr 5 Unknown

AAGCATAAAGTGTAAAGCCT**ACCCTAACCCTAACCCTAACCCTAACCCTAACCCT**GATTGCCATCACAGACAGGGG

AAGCATAAAGTGTAAAGCCTGGGGTGCCTAATGAGTGAGCTAACTCACATTAATTGCGTTGCGCTCACTG

TGAGCCCTCTGCACATGGCGCACCACCGTGTTTGCAAGACACAAGCCAGATTGCCATCACAGACAGGGG

Wildtype Chr 5 Primed RC

CGGCTGGCCGCGCAGCAACAGATGGAAGGCCTCCT**TGGGCCGGT**TACCTGGAGGTAAACCCACGTTCCTACGGGGT

CGGCTGGCCGCGCAGCAACAGATGGAAGGCCTCCTGGCGCCGCACCGGCCCAAGGAGCCCGCGTGGTTCCTGGCCAGGAAGATTGGCCATGAGAGGGCAGCAGCGAGTACGTACAAGCCGTACCTGGAGGTAAACCCACGTTCCTACGGGGT

Wildtype Chr 6 Unknown

GGAAGCATAAAGTGTAAAGCCTGGGGTGCCTAAT**ATGTGAGTT**AGCTTAATCCTACCATTGTGTACCTGCAGTTGC

GGAAGCATAAAGTGTAAAGCCTGGGGTGCCTAATGAGTGAGCTAACTCACATTAATTGCGTTGCGCTCACTGCCCG

CTAGGGTTAGCTCTTCAGCTGCAAATTCTGTGATGGAGAATTCAGCTTAATCCTACCATTGTGTACCTGCAGTTGC

Wildtype Chr 7 Unknown

CCGCCTGCAGGTCGACCATATGGGAGAGCTCCCAAC**CCATT**GGAAGGCGGGATTCAGCGCAAGACCCTGCTTCCTG

CCGCCTGCAGGTCGACCATATGGGAGAGCTCCCAACGCGTTGGATGCATAGCTTGAGTATTCTATAGTGTCACCTA

TTTGAGGGCGGAAGTGCGGGCGAAGTTGAGCGCGCACGAAAGGAAGGCGGGATTCAGCGCAAGACCCTGCTTCCTG

Wildtype Chr 9 Primed RC

TGAGCTAACTCACATTAATTGCGTTGCG**GGCAGTGAGCGGTTGCAAGGAC**GTTTCTCATTTTCAATGATTTTCATT

TGAGCTAACTCACATTAATTGCGTTGCGCTCACTGCCCGCTTTCCAGTCGGCTTAATGCGCCGCTACAGGGCGCGT

TTTCAGTGATTTCGTCATTTTTCACGTCGTCAAGTGGATGTTTCTCATTTTCAATGATTTTCATT

Wildtype Chr 9 Unknown

CACACAACATACGAGCCGGAAGCATAAAGT**ATAAAGTAAAGTTGGA**ATTTCTGAGTTCGAGGCCATCATGGTCTAT

CACACAACATACGAGCCGGAAGCATAAAGTGTAAAGCCTGGGGTGCCTAATGAGTGAGCTAACTCACATTAATTGC

TGGCGCACGCCTTTAATCCCAACACTCAGGAGGCAGAGGCAGGTGGATTTCTGAGTTCGAGGCCATCATGGTCTAT

Wildtype Chr 10 Unknown

TAGCTTGGCGTAATCATGGTCATAGCTGTTTCCTGTGTG**G**AATAAAGTTTGAAGACAGCTAGGAAGTTTATGTCCC

TAGCTTGGCGTAATCATGGTCATAGCTGTTTCCTGTGTGAAATTGTTATCCGCTCACAATTCCACACAACATACGA

AATTACAATGTACTAGCAGGAAAAAATAAATGTATGAACCAATAAAGTTTGAAGACAGCTAGGAAGTTTATGTCCC

Wildtype Chr 10 Primed RC

CCTAATGAGTGAGCTAACTCACATTAATTGCGTTGCG**GGCA**TTGCTTTAGGATGGAATGTTCTATAAATATGTTA

CCTAATGAGTGAGCTAACTCACATTAATTGCGTTGCGCTCACTGCCCGCTTTCCAGTCGGCTTAATGCGCCGCTA

GGAGAAGGTACCATGAGGTGCTGACAAGAAGGCATATTCTTTTGCTTTAGGATGGAATGTTCTATAAATATGTTA

Wildtype Chr 11 Unknown

AGTGAGCTAACTCACATTAATTGCGTTGCGC**AACTGTTGGGAAGG**GCAATACGTTAAAGTTGAATTCTGACCGTGG

AGTGAGCTAACTCACATTAATTGCGTTGCGCTCACTGCCCGCTTTCCAGTCGGCTTAATGCGCCGCTACAGGGCGC

TGACGGTCAGTAGGAAGGGTAAACGGACTGATTATGGATCGCATTGCAATACGTTAAAGTTGAATTCTGACCGTGG

Wildtype Chr 11 Unknown

AGCTGTTTCCTGTGTGAAATTGTTATCCGCTCACAATTC**A**CAACTGCTTCTGCCTAAACTCCCTGATGGTTCCTCA

AGCTGTTTCCTGTGTGAAATTGTTATCCGCTCACAATTCCACACAACATACGAGCCGGAAGCATAAAGTGTAAAGC

CTTCAATCTCTACCTCCTCATCTATCCAAAACACCAAATCCAACTGCTTCTGCCTAAACTCCCTGATGGTTCCTCA

Wildtype Chr 11 Primed RC

TCATGGTCATAGCTGTTTCCTGTGTGAAATTGT**GAGCGGATA**ACTGAGTCTGGGCTATAGGATCCAGGAATAAGA

TCATGGTCATAGCTGTTTCCTGTGTGAAATTGTTATCCGCTCACAATTCCACACAACATACGAGCCGGAAGCATA

TTTAACAAGGTTGGTCCTTGGATTTTAGGGCCCTGCTCTGAGACTGAGTCTGGGCTATAGGATCCAGGAATAAGA

Wildtype Chr 11 Primed RC

ATAGCTGTTTCCTGTGTGAAATTGT**GAGCGGATAACAATTATCGTAAGT**GGCAATGGGGAACGTCTCAACTGAGT

ATAGCTGTTTCCTGTGTGAAATTGTTATCCGCTCACAATTCCACACAACATACGA

GGTGTCAACTCTGCTAGAACTGGAGTTACAGGCAATGGGGAACGTCTCAACTGAGT

Wildtype Chr 11 Unknown

CGTATGTTGTGTGGAATTGTGAGCGGATAACAAT**ATCGTAAGT**GGCAATGGGGAACGTCTCAACTGAGTTCTGGGA

CGTATGTTGTGTGGAATTGTGAGCGGATAACAATTTCACACAGGAAACAGCTATGACCATGATTACGCCAAGC

CTTGTGGAGGCCAGGTGTCAACTCTGCTAGAACTGGAGTTACAGGCAATGGGGAACGTCTCAACTGAGTTCTGGGA

Wildtype Chr 12 Unknown

ACTCACATTAATTGCGTTGCGCTCACTGCCCGCTTTCCA**A**GGAACATTGTGAGCCTGTGTTGAGTTATCTGTAGCC

ACTCACATTAATTGCGTTGCGCTCACTGCCCGCTTTCCAGTCGGCTTAATGCGCCGCTACAGGGCGCGTCCATTCG

TGGCTAGTTAACCTTCAGGATACAGACTAGAATGGGACCCGGAACATTGTGAGCCTGTGTTGAGTTATCTGTAGCC

Wildtype Chr 13 Primed RC

CCTAATGAGTGAGCTAACTCACATTAATTGCGTTGCG**GGCAG**TCAGCTTGGGCTTCTGAAATATCATTTTGTTTTG

CCTAATGAGTGAGCTAACTCACATTAATTGCGTTGCGCTCACTGCCCGCTTTCCAGTCGGCTTAATGCGCCGCTAC

TTGCCAGATAAATACTCCACTCTTGGACCACACCCCCAACCCTCAGCTTGGGCTTCTGAAATATCATTTTGTTTTG

Wildtype Chr 17 Unknown

ACCGGGTAGGGGAGGCGCTTTTCCCAAGGCAGTCTG**CATTCC**AGGAAGAATCAAGATGTAAGGGGAAGCCAAAGCC

ACCGGGTAGGGGAGGCGCTTTTCCCAAGGCAGTCTGGAGCATGCGCTTTAGCAGCCCCGCTGGGCACTTGGCGCTA

TCTTTCCAGCCCCTAAGTTTTGTTTTGAATGACGCCATTAGAAGGAAGAATCAAGATGTAAGGGGAAGCCAAAGCC

Wildtype Chr 19 Primed RC

AGCATAAAGTGTAAAGCCTGGGGTGCCTAATGAGTGAG**T**TGCCTGGGGCTACCACTAGGAGGCTAATGAAAAGGGA

AGCATAAAGTGTAAAGCCTGGGGTGCCTAATGAGTGAGCTAACTCACATTAATTGCGTTGCGCTCACTGCCCGCTT

GCTTCAGGTCTGGAGAAACGCTAACAGACTCCCGTGTCCTGCCTGGGGCTACCACTAGGAGGCTAATGAAAAGGGA

Wildtype Chr X Primed RC

AACTCACATTAATTGCGTTGCG**GCTTAATGGGCAGTGAGCGCAACACAATTATATA**CTTCAGCTCCTTGGGTACTT

AACTCACATTAATTGCGTTGCGCTCACTGCCCGCTTTCCAGTCGGCTTAATG

TTGTTGTTCCACCTATAGGGTTGCAGACCCCTTCAGCTCCTTGGGTACTT

POLQ Chr 3 Unknown

TCGTGCAGATGGACAGCACCGCTGAGCAATGGAAGCGG**TTAG**GGACAGAAGAGCCATGTTTGAATTTGAATTTAAT

TCGTGCAGATGGACAGCACCGCTGAGCAATGGAAGCGGGTAGGCCTTTGGGGCAGCGGCCAATAGCAGCTTTGCTC

CATTCTTCTCGGTGGTGTACACATCTGTCCCAGTGTGGGTAAGGACAGAAGAGCCATGTTTGAATTTGAATTTAAT

POLQ Chr 3 Unknown

CTGCCCGCTTTCCAGTCG**GGAAACCTGTCGTGCCAGCTGCATTAATGAATCGGCCAACGCG**ATGTAGCCCAGCCTA

CTGCCCGCTTTCCAGTCGGCTTAATGCGCCGCTACAGGGCGCGTCCATTCGCCATTCAGGCTGCGCAA

TCAAAGGGTCACAGCATCAGGAAGGTTGGGAACCATTGTCCTACTGTCTCATGTAGCCCAGCCTA

POLQ Chr 5 Unknown

GTTGCGCTCACTGCCCGCTTTCCAGTCG**GGAAACCTGTCGTGCCAGCTGCATTA**AATTATATGTAATCAAAAATAC

GTTGCGCTCACTGCCCGCTTTCCAGTCGGCTTAATGCGCCGCTACAGGGCGCGTCCATTCGCCATTCAGGCTGCGC

GTTGATTGTTACATTTCTGAATATGTTAGAATATTATGGGTTTCCAATTTAATTATATGTAATCAAAAATACAC

POLQ Chr 14 Unknown

ACGCCGGAGAGCGTCGAAGCGGGGGCGGTGTTCGCCGAGA**C**ATTAACCCAGGGTAGCTCAAGCATGAAAGGGCTTT

ACGCCGGAGAGCGTCGAAGCGGGGGCGGTGTTCGCCGAGATCGGCCCGCGCATGGCCGAGTTGAGCGGTTCCCGGC

GCAGTCTCCGTTTCCTGTTCATCAACTTCCGAGATTCATGTATTAACCCAGGGTAGCTCAAGCATGAAAGGGCTTT

POLQ Chr 14 Unknown

GCTCACTGCCCGCTTTCCAGTCG**GGAAACCTGTCGT-/493/-TGTGCACGAACCC**ACATTTCCAATGCTATACCA

GCTCACTGCCCGCTTTCCAGTCGGCTTAATGCGCCGCTACAGGGCGCGTCCATTCGCCATTCAGGCTGCGCAA

TTCTGTCTTTTTTTTTTCTTTCCATTTTTTATTAGGTGTTTAGCTCATTTACATTTCCAATGCTATACCA

POLQ Chr 16 Unknown

CTGCCCGCTTTCCAGTCG**GAGGCTGGAGAGATGACTCATCCAGAGGCTGGAGAGATGACTCAT**CCAGAGGCTGGAG

CTGCCCGCTTTCCAGTCGGCTTAATGCGCCGCTACAGGGCGCGTCCATTCGCCATTCAGGCTGCGCAA

AAAGCATGGATTTTAAACACCTGAAATGTGGATTCTAAAGATGTCAAAACCCAGAGGCTGGAGAG

POLQ Chr 16 Unknown

TAAAGCCTGGGGTGCCTAATGAGTGAGCTAACTC**GCAGTGAGCGCAA**ATATTGATAGTCATTTCTCTCTCTCACTG

TAAAGCCTGGGGTGCCTAATGAGTGAGCTAACTCACATTAATTGCGTTGCGCTCACTGCCCGCTTTCCAGTCGGCT

GTGTATTCTCAGGAGAAATTAAGGTAGTTGCATAGCTTGAGGTCTGCATATTGATAGTCATTTCTCTCTCTCACTG

POLQ Chr 19 Unknown

TGGGGTGCCTAATGAGTGAGCTAACTCACATTAATTGCGT**G**TGGGGTAGCTTGGTTTTCCTTGGCAGATTACTAAC

TGGGGTGCCTAATGAGTGAGCTAACTCACATTAATTGCGTTGCGCTCACTGCCCGCTTTCCAGTCGGCTTAATGCG

TCTGCCCTGGTCACGTGCTCAGCAGCATACGTTCGATGTTTTGGGGTAGCTTGGTTTTCCTTGGCAGATTACTAAC
